# Novel agents for anti-platelet therapy

**DOI:** 10.1186/1756-8722-4-44

**Published:** 2011-11-04

**Authors:** Xuebin Ji, Ming Hou

**Affiliations:** 1 Department of Hematology, QiLu Hospital of Shandong University, Jinan, China PR

**Keywords:** anti-platelet, agent, therapy, antagonist, thrombotic disease

## Abstract

Anti-platelet therapy plays an important role in the treatment of patients with thrombotic diseases. The most commonly used anti-platelet drugs, namely, aspirin, ticlopidine, and clopidogrel, are effective in the prevention and treatment of cardio-cerebrovascular diseases. Glycoprotein IIb/IIIa antagonists (e.g., abciximab, eptifibatide and tirofiban) have demonstrated good clinical benefits and safety profiles in decreasing ischemic events in acute coronary syndrome. However, adverse events related to thrombosis or bleeding have been reported in cases of therapy with glycoprotein IIb/IIIa antagonists. Cilostazol is an anti-platelet agent used in the treatment of patients with peripheral ischemia, such as intermittent claudication. Presently, platelet adenosine diphosphate P2Y(12) receptor antagonists (e.g., clopidogrel, prasugrel, cangrelor, and ticagrelor) are being used in clinical settings for their pronounced protective effects. The new protease-activated receptor antagonists, vorapaxar and atopaxar, potentially decrease the risk of ischemic events without significantly increasing the rate of bleeding. Some other new anti-platelet drugs undergoing clinical trials have also been introduced. Indeed, the number of new anti-platelet drugs is increasing. Consequently, the efficacy of these anti-platelet agents in actual patients warrants scrutiny, especially in terms of the hemorrhagic risks. Hopefully, new selective platelet inhibitors with high anti-thrombotic efficiencies and low hemorrhagic side effects can be developed.

## Introduction

**Figure 1:**
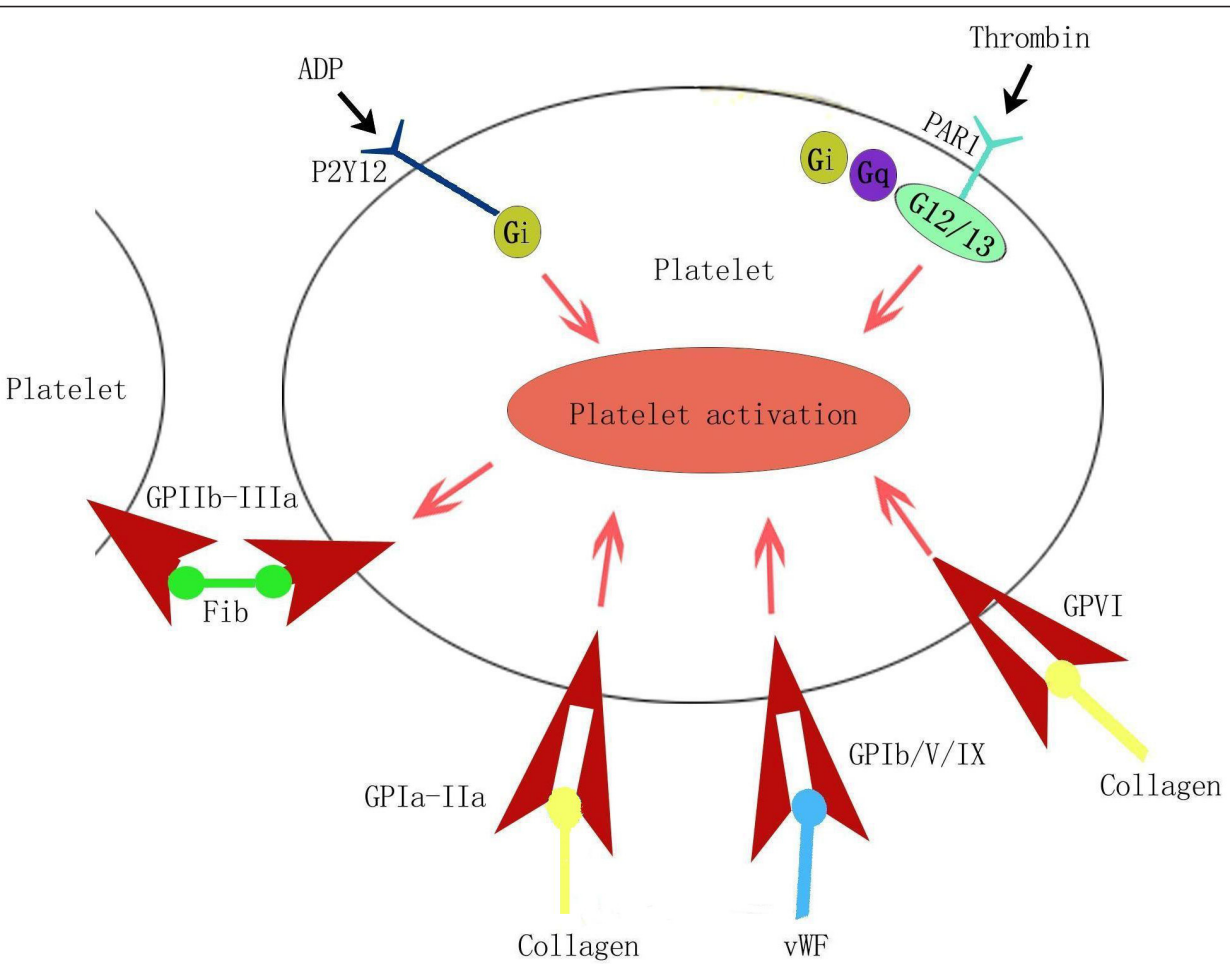
**Different targets for anti-platelet therapy**. According to the different targets, novel anti-platelet agents with different mechanism of action can be developed, including GP IIb/IIIa antagonists, P2Y(12) receptor antagonists and Protease-activated receptor antagonists, etc.

Thrombotic diseases and their complications may have severe consequences. Platelets play a key role in thrombosis, and anti-platelet therapies may prevent as well as treat thrombotic diseases. Therefore, anti-platelet drugs that can inhibit platelet adhesion, aggregation, release, and activation need to be developed (Figure 1). The most commonly used anti-platelet drugs, namely, aspirin, clopidogrel, and ticlopidine are effective in preventing thrombotic diseases. With the developments in medicine and pharmacy, the number of anti-platelet agents is continuously increasing.

## Platelet glycoprotein (GP) IIb/IIIa receptor antagonists (Table 1)

The adhesion, aggregation, release, and activation of platelets can induce platelet thrombosis, which is important in physiological hemostasis and pathological thrombosis. Once platelets are activated, GP IIb/IIIa receptors on the surfaces of platelets transform into their active states, which can combine with fibrinogen and the von Willebrand factor (vWF). The GP IIb/IIIa receptor operates in the final common pathway of platelet aggregation. Blocking the GP IIb/IIIa receptor can inhibit platelet aggregation induced by activating factors. Once platelet aggregation is inhibited, platelet thrombi cannot form.

**Table 1:** Glycoprotein IIb/IIIa antagonists

Agents	Mechanism of action	Administration	Main side effects	State
Abciximab	inhibit GPIIb/IIIa receptor and GP αIIb/β3 receptor	IV	allergy, bleeding, thrombocytopenia	Approved
Eptifibatide	inhibit GPIIb/IIIa receptor	IV	bleeding, thrombocytopenia	Approved
Tirofiban	inhibit GP IIb/IIIa receptor	IV	bleeding, thrombocytopenia	approved

Abbreviations: GP: glycoprotein; IV: intravenous.

The development of GP IIb/IIIa antagonists, such as the recently approved abciximab, eptifibatide, and tirofiban, is pivotal in anti-platelet therapy. Pharmacodynamic studies on these three agents have revealed their capabilities of establishing and maintaining a > 80% inhibition of platelet aggregation [1].

The first GP IIb/IIIa receptor antagonist used in clinical settings is abciximab. This drug is the fragment of recombinant human-mouse chimeric monoclonal antibody, which can inhibit GP IIb/IIIa receptors in a dose-dependent manner. Abciximab also inhibits αIIb/β3 receptors (for vWF) on platelets, thereby inhibiting platelet aggregation via fibrinogen. However, abciximab have the disadvantages of potential immunogenicity, drug effect irreversibility, and high cost [2]. Hence, micromolecular GP IIb/IIIa receptor antagonists (e.g., eptifibatide and tirofiban) have been developed. These micromolecular GP IIb/IIIa receptor antagonists contain the Arg-Gly-Asp (RGD) sequence. In the RGD sequence of eptifibatide, an arginine residue is replaced by the lysine residue. On the other hand, tirofiban is the micromolecular GP IIb/IIIa receptor antagonist synthesized according to the RGD module. These micromolecular agents, unlike abciximab, specifically act on GP IIb/IIIa receptors and do not combine with any other integrin. Eptifibatide and tirofiban also cannot induce immune response given their small molecular weights and low affinities to GP IIb/IIIa receptors.

Abciximab, eptifibatide, and tirofiban are all intravenously injected. Large-scale clinical trials have demonstrated the clear clinical effects and safety of these drugs in decreasing the ischemic events in acute coronary syndrome (ACS). Their uses in adjunctive therapy during percutaneous coronary intervention (PCI) have also been revealed [3,4]. However, adverse events related to thrombosis or bleeding have still been reported in cases of therapy with GP IIb/IIIa [5]. Trials on orally administered GP IIb/IIIa antagonists have failed to demonstrate any benefit, and even indicated significantly increased mortality in ACS cases [6]. Therefore, the development of GP IIb/IIIa antagonists needs further functional testing methods to assess the anti-platelet efficacy of these drugs in actual patients.

## Agents that selectively increase cyclic adenosine 3'-5'-monophosphate (cAMP) in platelets

Platelet aggregation can be inhibited either by the blocking of membrane receptors or interaction with intracellular signaling pathways. cAMP and cyclic guanosine 3'-5-monophosphate (cGMP) are two important intracellular second messengers for platelet function. Phosphodiesterase (PDE), which is obtained by catalyzing the hydrolysis of cAMP and cGMP, limits the intracellular levels of cyclic nucleotides to regulate platelet function. Therefore, the inhibition of PDEs may confer a strong inhibitory effect on platelets.

Cilostazol is an anti-platelet agent used in the treatment of peripheral ischemia, such as intermittent claudication. This drug is a kind of selective inhibitor of cAMP-PDE that can dilate blood vessels and hinder platelet aggregation induced by adenosine diphosphate (ADP), collagens and arachidonic acid. Unlike aspirin, cilostazol is a reversible platelet inhibitor that prevents both primary and secondary aggregation. In terms of pharmacokinetics, cilostazol is metabolized in the liver and excreted by the kidney.

Overall, cilostazol is a good drug of choice given its high tolerance, small number of side effects, high safety, and potential use with other anti-platelet drugs [7-9]. Cilostazol may also be a more safer and effective alternative to aspirin in the patients with ischemic stroke [10]. Presently, there is a trend towards enhanced anti-platelet effects when cilostazol is added to aspirin in the ischemic stroke patients. A combination of aspirin and cilostazol may be a good treatment option for these patients [11,12].

## P2Y(12) receptor antagonists(Table 2)

P2Y(12) receptor antagonists are anti-thrombotic agents that inhibit platelet function by blocking the ADP at P2Y(12) receptor sites. Adenine nucleotides act on platelets via three distinct P2 receptors, namely, two G protein-coupled ADP receptors, P2Y(1) and P2Y(12), as well as a P2X(1) receptor ligand-gated cation channel activated by adenosine triphosphate (ATP). The P2Y(1) receptor initiates platelet aggregation, but is not sufficient in response to ADP. On the other hand, the P2Y(12) receptor is responsible for the completion of aggregation in response to ADP. The P2Y(12) receptor is the molecular target of anti-thrombotic drugs such as clopidogrel, prasugrel, cangrelor, and ticagrelor. This receptor is responsible for most of the potentiating effects of ADP when platelets are activated by agonists such as collagen, thrombin [13,14]. These platelet antagonists blocking the ADP-receptor P2Y(12) decrease myocardial infarction, stroke, thrombosis and mortality in the patients with cardiovascular diseases. The P2Y(12) antagonists (e.g., clopidogrel, prasugrel, and ticagrelor) are now being used in clinical settings given their more protective effects [15].

**Table 2:** P2Y(12) receptor antagonists

Agents	Mechanism of action	Administration	Main Side effects	State
Clopidogrel	thienopyridine, blocking the effects of ADP at P2Y(12) receptor sites	Oral	bleeding, thrombocytopenia	approved
Prasugrel	thienopyridine, blocking the effects of ADP at P2Y(12) receptor sites	Oral	bleeding	approved
Cangrelor	nonthienopyridine, the blocking effects of ADP at P2Y(12) receptor sites	IV	bleeding	unapproved
Ticagrelor	nonthienopyridine, the blocking effects of ADP at P2Y(12) receptor sites	Oral	bleeding	approved

Abbreviations: IV: intravenous; ADP:adenosine diphosphate.

Presently, the P2Y(12) receptor antagonists being developed are clopidogrel and prasugrel, which are thienopyridines, as well as cangrelor and ticagrelor, which are nonthienopyridines. Hence, these antagonists are potential anti-thrombotic drugs.

Clopidogrel is a thienopyridine with proven antithrombotic efficacy. However, it has the disadvantages of high inter-individual variability in pharmacological response, delayed onset and offset of action, as well as requiring to be metabolized to its active metabolite form [16].

Prasugrel has received its priority right of approval in 2008. It is a new kind of thienopyridine P2Y12 receptor antagonist that works by blocking the P2Y12 ADP receptor on the surfaces of platelets. Nevertheless, the clinical benefits of prasugrel are countered by increased bleeding risk compared with the conventional thienopyridine treatment using clopidogrel. In the clinical trial of prasugrel versus clopidogrel in patients with acute coronary syndromes (ClinicalTrials.gov number, NCT00097591), the rates of myocardial infarction in the prasugrel group were significantly reduced (9.7% for clopidogrel vs. 7.4% for prasugrel; P < 0.001). There were also significant reductions in the urgent target-vessel revascularization (3.7% vs. 2.5%; P < 0.001), and stent thrombosis (2.4% vs. 1.1%; P < 0.001). However, there was more significant major bleeding in patients receiving prasugrel (2.4%) than in patients receiving clopidogrel (1.8%) (P = 0.03). The prasugrel group also had greater rate of life-threatening bleeding (1.4% vs. 0.9%; P = 0.01), including nonfatal bleeding (1.1% vs. 0.9%; P = 0.23) and fatal bleeding (0.4% vs. 0.1%; P = 0.002)[17].

Still, some current practice guidelines incorporate prasugrel as a treatment option because it has a faster onset of action and more uniform inhibition of platelet function than clopidogrel. However, prasugrel is not presently recommended to be selected over clopidogrel in any patient subgroup. Further studies are required to determine the optimal dosage and patient characteristics for prasugrel treatments [18].

Two direct and reversible P2Y(12) antagonists, cangrelor and ticagrelor, are characterized by rapid onsets and reversibilities of platelet inhibition.

Similar with the nonthienopyridine P2Y(12) receptor antagonist, cangrelor is a reversible anti-platelet intravenous preparation with a rapid effect because it can function without needing to be metabolized. In a study of PCI patients, the use of cangrelor has shown no significant difference compared with abciximab in major adverse cardiac events and bleeding complications. Cangrelor is only intravenously administrated. However, there is no evidence that can prove its superiority over current commonly used intravenous anti-platelet agents (e.g., GP IIb/IIIa receptor antagonists such as abciximab) in preventing thrombotic events in patients undergoing PCI. Indeed, its therapeutic potential is uncertain [19].

On the other hand, ticagrelor is better than clopidogrel in preventing major adverse cardiac events in ACS patients. Compared with clopidogrel, ticagrelor decreases the incidences of major adverse cardiac events and thrombosis. Similar with prasugrel, ticagrelor is associated with high frequencies of bleeding complications. A short period of drug discontinuation before surgery is generally necessary in the ticagrelor-treated patients in order to limit the risk of post-surgical bleeding [20-23].

## Protease-activated receptor (PAR) antagonists (Table 3)

PARs include PARA1, PARA2, PARA3, and PARA4. Similar with thrombin receptors, PAR1, PAR3, and PAR4 mediate platelet activation induced by thrombin. In humans, only PAR1 and PAR4 are expressed. The ADP and thromboxane A2 (TXA2) platelet activation pathways exist during the progress of pathological thrombosis and physiological hemostasis. The combined use of aspirin and clopidogrel well inhibits thrombosis in induced hemorrhagic complications. PAR1-mediated platelet activation mainly promotes pathological thrombosis and has less influence on protective hemostasis. Hence, PAR1 antagonists decrease the incidence of hemorrhagic complications [24,25]. Oral PAR1 antagonists currently being researched include vorapaxar (SCH 530348) and atopaxar (E-5555).

**Table 3:** Protease-activated receptor (PAR) antagonists

Agents	Mechanism of action	Administration	Main side effects	State
Vorapaxar	a non-peptide competitivePAR1 thrombin receptor antagonist with a high affinity and low molecular weight	oral	bleeding	unapproved
Atopaxar	PAR1 antagonist inhibiting thrombin-mediated platelet aggregation	oral	bleeding	unapproved

Abbreviations: PAR:protease-activated receptor.

Vorapaxar, a synthetic tricyclic 3-phenylpyridine, is a new orally active fourth generation himbacine-based antagonist of the protease-activated receptor PAR1, and is the primary receptor for thrombin in human platelets. Vorapaxar is a non-peptide competitive PAR1 thrombin receptor antagonist with a high affinity and low molecular weight. It belongs to the first agent in a new type of compounds that inhibit thrombin-mediated platelet aggregation without affecting the enzymatic activity of thrombin on fibrinogen. Preclinical and initial clinical studies have demonstrated the high potential of vorapaxar in inhibiting thrombin-induced platelet activation, as well as its excellent oral bioavailability and safety [26]. Vorapaxar inhibits thrombin-mediated platelet aggregation mediated, and does not influence hemostasis as well as bleeding time. Hence, this drug potentially decreases the risk for ischemic events without significantly increasing the rate of bleeding. Vorapaxar, which is orally administered, is rapidly absorbed and has a long biological half-life [27,28].

Atopaxar is another powerful oral PAR1 antagonist. Preclinical studies have indicated that atopaxar inhibits thrombin-mediated platelet aggregation mediated without increasing the rate of bleeding. The selective blocking of platelet receptors by atopaxar suggests its therapeutic potential in thrombotic disease [29-31].

## Novel anti-platelet drugs

The interaction between platelets and collagen is the motivator of platelet adhesion, aggregation, and activation. This interaction has become the target for developing new anti-platelet agents. On the surfaces of platelets, there exist at least three kinds of receptors that can combine with collagen, including GP Ib/IX (functions with the vWF), GP Ia-IIa (the main receptor in platelet adhesion), and GPVI (mediates platelet activation). Platelets adhesion to the damaged blood vessel is the initial trigger for arterial hemostasis and thrombosis. Platelets adhere to the sub-endothelium via their interaction with the vWF, which forms a bridge between collagens within the damaged vessel wall [32-34]. The development of monoclonal antibodies that inhibit platelet adhesion or aggregation targets collagen-vWF-GPIb and collagen-GPVI [35-37].

The 6B4-antigen-binding fragment (Fab) is a murine monoclonal antibody that targets the human platelet GPIb alpha (201-268aa) and blocks the binding of the vWF. It has been proved as a powerful anti-thrombotic agent without the side effects of bleeding or thrombocytopenia. The anti-thrombotic effects of 6B4-Fab on acute platelet-mediated thrombosis have been studied in baboons. Minimal effects on the bleeding time, absence of spontaneous bleeding, and absence of thrombocytopenia are observed. Hence, H6B4-Fab can be further developed [38]. There are some other promising preclinical agents targeting GP Ib. Such agents include GPG-290, a recombinant chimeric protein containing amino-terminal 290 amino acids of alpha linked to the human IgG1 crystallizable fragment, and Shuzhou2 (SZ2), an anti-GP Ib monoclonal antibody [39].

The vWF plays an important role in both hemostasis and thrombosis. Platelets adhere to damaged arteries by the interactions between the vWF A1-domain and glycoprotein Ib receptors under conditions of high shear. This initial platelet binding event stimulates platelet activation, recruitment, and activation of the clotting cascade, thereby promoting thrombus formation. Therefore, the monoclonal antibody against the functional domains (A1 and A3) of the vWF can effectively inhibit thrombosis. The genetically engineered antibody AJW200 is a humanized anti-vWF-A1 chimeric Fab antibody that can block the combination of the vWF and GP Ib. Consequently, platelet adhesion and aggregation are inhibited without remarkably prolonging the bleeding time. The recombinant chimeric monoclonal antibody 82D6A3 against the vWF-A3 region can block the combination of the A3 region and collagen to inhibit platelet adhesion. ARC1779, which has a high binding affinity with the vWF A1-domain, has been proven to inhibit botrocetin-induced and shear force-induced platelet aggregation. Besides, ARC15105 is a chemically advanced follower with a potential higher affinity to the vWF [40,41].

GPVI, which is a major receptor of collagen on platelet surfaces, mediates the initial platelet contacting with collagen, which can cause platelet adhesion, aggregation, and thrombosis [42,43]. GPVI may be considered as an interesting and prospective target in the development of anti-platelet agents [44-46]. Anti-GPVI antibodies, such as the monoclonal antibody JAQ1, can significantly prevent thrombosis with a little prolonged bleeding time [47].

## Conclusions and future directions

Given the increasing incidence of and mortality from thrombotic diseases, anti-platelet agents have been extensively researched and developed [48,49]. The combined use of anti-platelet drugs with different mechanisms may be important in anti-thrombotic treatments. Further research on platelet functions may give rise to numerous new anti-platelet agents. Pharmacodynamic platelet function assays and pharmacokinetic tests for individualizing and optimizing anti-platelet therapy may find their way into clinical use. However, more studies are needed. Hopefully, new selective platelet inhibitors with high anti-thrombotic efficiencies and low adverse hemorrhagic side affects can be developed.

## List of abbreviations used

GP: glycoprotein; vWF: von Willebrand factor; PCI: percutaneous coronary intervention; ACS: acute coronary syndrome; RGD: Arg-Gly-Asp; ADP: adenosine diphosphate; ATP: adenosine triphosphate; cAMP: cyclic adenosine 3'-5'-monophosphate; cGMP: cyclic guanosine 3'-5-monophosphate; PDE: phosphodiesterase; PAR: protease-activated receptor; TXA2: thromboxane A2; Fab: antigen-binding fragment; SZ2: shuzhou2

## Competing interests

The authors declare that they have no competing interests.

## Authors' contributions

The present manuscript was drafted by XJ and revised by MH. All authors read and approved the final manuscript.
